# Impacts of tissue-type plasminogen activator (tPA) on neuronal survival

**DOI:** 10.3389/fncel.2015.00415

**Published:** 2015-10-16

**Authors:** Arnaud Chevilley, Flavie Lesept, Sophie Lenoir, Carine Ali, Jérôme Parcq, Denis Vivien

**Affiliations:** INSERM, UMR-S U919 Serine Proteases and Pathophysiology of the Neurovascular Unit, Université Caen-NormandieCaen, France

**Keywords:** tissue-type plasminogen activator, excitotoxicity, apoptosis, NMDA receptors, differential effects

## Abstract

Tissue-type plasminogen activator (tPA) a serine protease is constituted of five functional domains through which it interacts with different substrates, binding proteins, and receptors. In the last years, great interest has been given to the clinical relevance of targeting tPA in different diseases of the central nervous system, in particular stroke. Among its reported functions in the central nervous system, tPA displays both neurotrophic and neurotoxic effects. How can the protease mediate such opposite functions remain unclear but several hypotheses have been proposed. These include an influence of the degree of maturity and/or the type of neurons, of the level of tPA, of its origin (endogenous or exogenous) or of its form (single chain tPA versus two chain tPA). In this review, we will provide a synthetic snapshot of our current knowledge regarding the natural history of tPA and discuss how it sustains its pleiotropic functions with focus on excitotoxic/ischemic neuronal death and neuronal survival.

## The Natural History Of tPa

Morgagni (1761) noted that the blood of patients who died suddenly was not completely coagulated. Denis (1838) observed the spontaneous dissolution of blood clots. Fifty years later, Denys and de Marbaix (1889) postulated the existence of an endogenous fibrinolytic enzyme. Accordingly, Hedin (1903) revealed a proteolytic activity in serum globulin fraction, later identified as the fraction containing a precursor of plasmin. Christensen and Macleod (1945) proposed that this inactive circulating precursor, named plasminogen, could be activated by bacterial extracts like streptokinase. Macfarlane and Biggs (1948) completed the description of the plasminogen activation cascade. In parallel, Conradi (1902) identified tPA, at this time named fibrikinase, in different organs), later characterized to mediate fibrinolysis (Fleisher and Loeb, 1915; Astrup and Permin, 1947; Astrup and Stage, 1952). tPA was then purified from human vessels and uterus in Binder et al. (1979), Rijken et al. (1979) and in larger amounts from Bowes melanoma cell line allowing its biochemical characterization (Collen et al., 1982; Collen and Lijnen, 2009). Pennica et al. (1983) succeeded in cloning and expressing recombinant tPA, providing the primary structure of tPA. tPA is a protein of 527 amino-acids including three glycosylation sites and 17 disulfide bridges (Pennica et al., 1983). Collen and Lijnen (1991) then provided evidence that tPA could facilitate the dissolution of blood clots by inducing the degradation of fibrin in a plasminogen-dependent manner. tPA is now used in the clinic to promote fibrinolysis, especially at the acute phase of ischemic stroke either alone (NINDS, 1995) or combined with thrombectomy (Campbell et al., 2015; Goyal et al., 2015).

In addition to this fibrinolytic function at the origin of its discovery, an increasing number of studies have since the mid-90s, discovered functions of tPA within the brain parenchyma. In particular, tPA is believed to control neuronal fate during several CNS disorders, including multiple sclerosis, Alzheimer’s disease, and stroke. The aim of this review is to summarize and discuss structure-function studies related to the influence of tPA on neuronal death and survival.

## tPA or tPAs?

The mature form of tPA is a mosaic protein of five distinct modules, which, from its N-terminal end to its C-terminal end, are: a finger domain (F), an epidermal growth factor-like domain (EGF), two kringle domains (K1 and K2), and a serine protease proteolytic domain (SP). The finger domain is involved in tPA binding to fibrin and is necessary to promote fibrinolytic activity at low plasminogen activator concentrations (Larsen et al., 1988). In the brain, other functions attributed to the finger domain include its ability to cross the blood–brain barrier (Benchenane et al., 2005), its astrocytic clearance (Cassé et al., 2012) and some of its signaling pathways (Siao and Tsirka, 2002; Pineda et al., 2012). The EGF-like domain shows homology with EGF. Both the trophic and mitogenic functions of tPA have been attributed to this domain (Liot et al., 2006; Ortiz-Zapater et al., 2007; Correa et al., 2011; Haile et al., 2012). The EGF-like domain has been also reported to contribute to the hepatic recapture of tPA (Hajjar and Reynolds, 1994). The kringle domains fold into large loops stabilized by three disulfide bridges. Because of the high-mannose-type glycosylation at Asn117, K1 is of major importance in the uptake of tPA by mannose receptors on liver endothelial cells *in vivo* and *in vitro* (Kuiper et al., 1996). The K2 domain and more specifically its lysine binding site (LBS) is involved in the capacity of tPA to bind and activate substrates and/or receptors such as plasminogen, PDGF-CC (platelet derived growth factor-CC; Fredriksson et al., 2004) and NMDAR (*N*-methyl-D-aspartate receptor; López-Atalaya et al., 2008). The K1 of tPA does not possess a LBS (Kim et al., 2003). The C-terminal domain supports the catalytic activity of tPA and forms the catalytic triad (*His 322, Asp 371, and Ser 478)* involving an aspartic acid residue (Asp371) hydrogen-bonded to a histidine (His322), which itself is hydrogen-bonded to a serine (Ser478).

As detailed here after, the literature suggests that there is not one but several forms of tPAs.

### Long and Short Variants

The pro-form of tPA is a molecule of 562 amino acids. The signal peptide and a pro-peptide of, respectively, 22 and 10 amino acids should be removed before storage in vesicles and release. Three additional amino acids (Gly–Ala–Arg) at the N-terminal end of the molecule can be also removed leading to the release of either the long variant (L-tPA) or the short variant (S-tPA) of 530 and 527 amino acids, respectively (Jörnvall et al., 1983; Berg and Grinnell, 1991). These tPAs include 17 disulfide bridges.

### sc-tPA vs. tc-tPA

**FIGURE 1:**
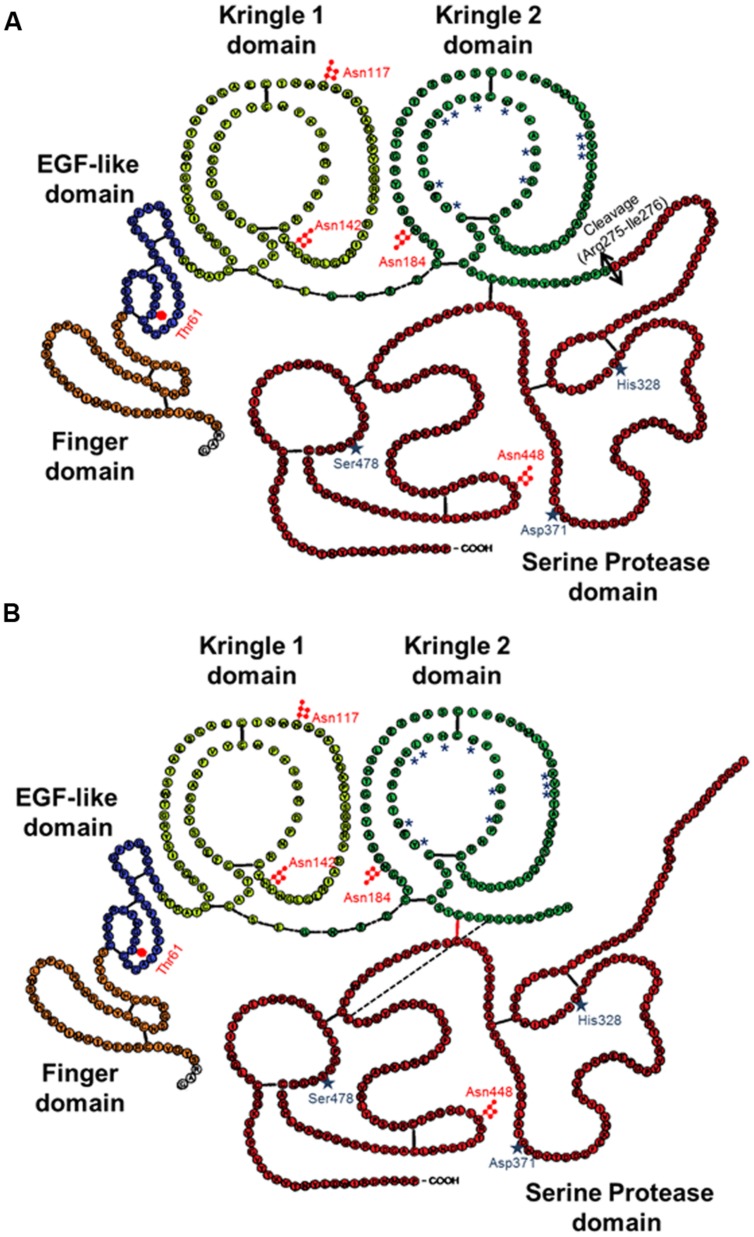
**Schematic representations of the primary structure of sc-tPA (A) and tc-tPA (B).** Each amino acid is represented by its single letter symbol. Sites of N- (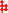) or O-glycosylation (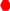) are showed. The active site residues His322, Asp371, and Ser478 are marked by stars. The amino acids involved in the structure of the lysine binding site are noted with asterisks. The black bars indicate disulfide bonds. The black bar dotted indicate salt bond. The double-arrow indicates the cleavage site for conversion of sc-tPA to tc-tPA.

In contrast to the other members of the chymotrypsin family, tPA is not synthesized and secreted as a “true” zymogen (Madison et al., 1993). Like other members of the family, the secreted single-chain tPA (sc-tPA; **Figure 1A**) can be processed into a two-chain form tPA (tc-tPA; **Figure 1B**) by plasmin or kallikrein (Wallén et al., 1982; Ichinose et al., 1984). However, sc-tPA is an unusually active zymogen (high intrinsic proteolytic activity, low zymogenicity) that does not require proteolytic processing to be active but relies on the presence of an allosteric regulator, such as fibrin (Thelwell and Longstaff, 2007). The passage from the sc-tPA to the tc-tPA form results from the hydrolysis of the peptide bond linking the Arg275 and the Ile276, both parts of the protein remaining connected by a disulfide bridge between Cys299 (heavy chain A) and Cys430 (light chain B) and a novel salt bridge between Arg302 and Glu445 (Lamba et al., 1996). In the absence of an allosteric regulator such as fibrin, tc-tPA is fivefold catalytically more active than sc-tPA (Rånby et al., 1982; Wallén et al., 1982; Tate et al., 1987; Petersen et al., 1988; Boose et al., 1989). However, in the presence of fibrin, both sc-tPA and tc-tPA display the same catalytic activity (Thelwell and Longstaff, 2007).

### Type I vs. Type II tPA

**FIGURE 2:**
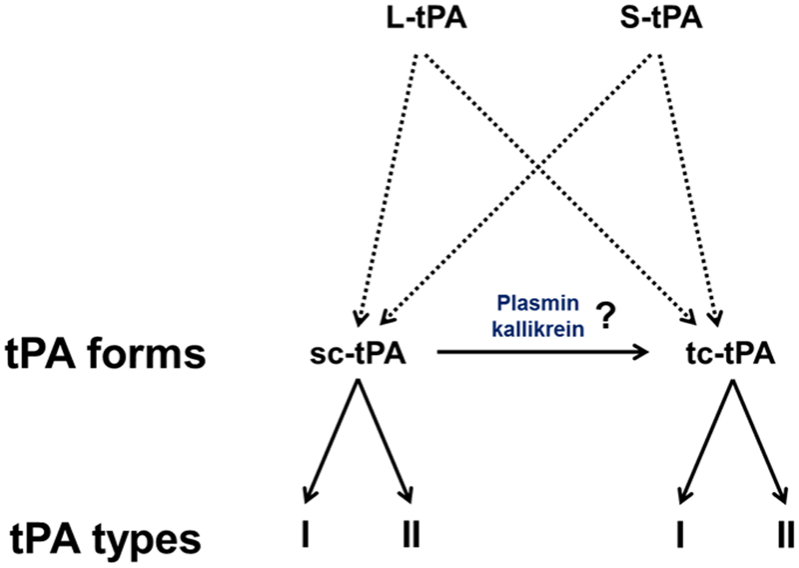
**The diversity of tPAs.** L-tPA and S-tPA are released under their single chain form (sc-tPA), possibly cleaved into their two-chain form (tc-tPA) by plasmin or kalikrein. Each form of tPA exists in two glycosylated states, types I or II.

Type plasminogen activator is a glycoprotein containing three major N-glycosylation sites. Two glycosylations are constitutives at Asn117 within the kringle 1 domain and at Asn448 within the serine protease domain. A third one is alternative at Asn184 within the kringle 2 domain. Type I tPA is glycosylated at Asn117, Asn184, and Asn448, while type II tPA is glycosylated only at Asn117 and Asn448 (Pohl et al., 1984; Spellman et al., 1989; Mori et al., 1995; Jaques et al., 1996). Asn184 acts as a switch that enables long-distance communication between fibrin-binding residues (achieved by the finger domain) and the catalytic site in the protease domain (Rathore et al., 2012). Glycosylation of Asn184 (i.e., type I) reduces the ability of tPA to activate plasminogen as well as its binding to fibrin (Einarsson et al., 1985; Wittwer et al., 1989; Berg et al., 1993). Type I sc-tPA seems to be more stable than type II sc-tPA regarding its conversion to tc-tPA (Wittwer and Howard, 1990; Berg et al., 1993; **Figure 2**). tPA also contains a O-linked fucose at Thr61 (occupancy 100%) within the EGF domain (Harris et al., 1991) and potentially an additional N-glycosylation site at Asn142 within the K1 domain (occupancy 1%; Borisov et al., 2009).

## Is tPA Good or Bad for Neuronal Survival?

### The Facts

**FIGURE 3:**
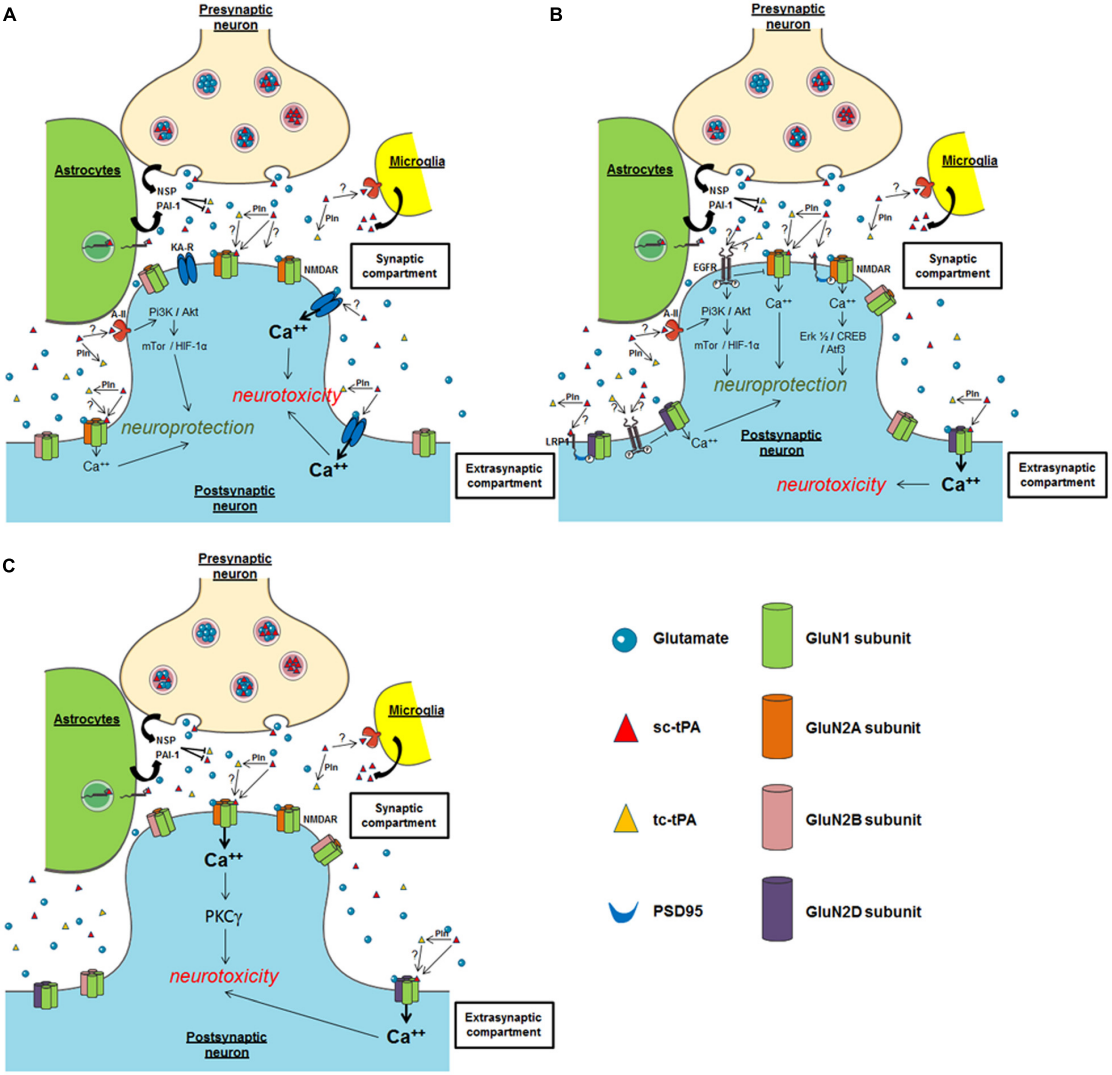
**Possible mechanisms of tPA on neuronal survival. (A)** Hippocampal neurons; **(B)** cortical neurons; **(C)** cerebellar neurons. NSP, neuroserpin; Pln, plasmin; PAI-1, plasminogen activator inhibitor-1; NMDAR, *N*-methyl-D-aspartate receptor; EGFR, epidermal growth factor receptor; KA-R, kainate receptor; A-II, annexin II receptor.

The group of Sidney Strickland was the first to demonstrate that tPA deficient mice were more sensitive to hippocampal neuronal death induced by both NMDAR- and non-NMDAR-agonists (Tsirka et al., 1995), an effect dependent of the ability of tPA to activate plasminogen into plasmin (Tsirka et al., 1997a,b; **Figure 3A**). Accordingly, several studies have reported that inhibitors of tPA, such as neuroserpin and type 1 plasminogen activator inhibitor (PAI-1) protect neurons against toxicity induced by the over-activation of NMDARs (Buisson et al., 1998; Zhang et al., 2002; Gabriel et al., 2003; Lebeurrier et al., 2005). Exogenous tPA was then reported pro-neurotoxic, on cortical neurons, in paradigms of *in vitro* or *in vivo* excitotoxicity mediated by over-activation of NMDAR (Nicole et al., 2001; Liberatore et al., 2003; Reddrop et al., 2005; Park et al., 2008; **Figure 3B**). The tPA was also reported to promote damages on Purkinje cells (Lu and Tsirka, 2002; Li et al., 2006, 2013; Cops et al., 2013; **Figure 3C**), especially by altering the neurotrophic mechanisms that control their postnatal development (Li et al., 2006, 2013).

Both plasmin-dependent and plasmin-independent mechanisms have been proposed to explain the potentiation of NMDAR signaling by tPA (Nicole et al., 2001; Pawlak et al., 2002; Matys and Strickland, 2003), but several recent studies agree that it can occur independently of plasminogen activation (Samson et al., 2008; Echeverry et al., 2010; Parcq et al., 2012). For instance, tPA can interact with the GluN1 subunit of NMDAR involving the LBS of its K2 domain (Nicole et al., 2001; Fernández-Monreal et al., 2004; Kvajo et al., 2004; López-Atalaya et al., 2008; Parcq et al., 2012). Our group reported that the cleavage of the amino-terminal domain of GluN1 subunit is necessary for enhancement of NMDAR signaling by tPA (Nicole et al., 2001; Fernández-Monreal et al., 2004). In the brain of protease nexin-1 (PN-1, an inhibitor of tPA) deficient mice, Kvajo et al. (2004), demonstrated an increase in the proteolytic activity of tPA, correlated with a decrease in the amount of the GluN1 subunit of the NMDA receptor. However, no cleavage of GluN1 was observed despite the interaction of tPA with the GluN1 subunits of NMDAR (Kvajo et al., 2004). Other groups did not detect tPA-dependent cleavage of GluN1, despite enhancement of NMDAR function by exogenous tPA in cortical cultures (Samson et al., 2008). In a more recent study, it was reported that sc-tPA, but not tc-tPA can promote NMDAR signaling and neurotoxicity in cortical neurons (Parcq et al., 2012; Bertrand et al., 2015). These data were the first to describe a differential function of sc-tPA and tc-tPA. tPA would also act on neuronal death by engaging Low density lipoprotein related protein (LRP) receptors, which in turn would enhance Ca^2+^ downstream of NMDAR (Samson et al., 2008). More recent data obtained from Schwann cells showed that tPA can promote NMDAR signaling independently of LRP1 (Mantuano et al., 2015). Similarly, in PC12 and N2a neuron-like cells, tPA may signal through a complex containing NMDAR, LPR1, and Trk receptors (Mantuano et al., 2013). Plasmin, which is generated by the tPA-dependent conversion of plasminogen, has also been reported to cleave NMDARs, specifically the GluN2 subunit. This cleavage can occur at two sites: Lys317 on GluN2A, which relieves Zn^2+^ inhibition and thereby increases NMDAR function (Yuan et al., 2009), and Arg67 on GluN2B, which increases sensitivity of the NMDAR to glycine (Ng et al., 2012). Whether tPA-dependent plasmin formation counteracts or interferes with tPA-dependent NMDAR activation is still under debate. Whatever the mechanism, all these studies showed that tPA can increase NMDAR signaling.

By contrast, other studies, in particular using transgenic mice over-expressing tPA in neurons (T4 transgenic mice) or tPA KO mice, suggested that tPA can also have neuroprotective effects (Haile et al., 2012; Wu et al., 2012). These two studies also proposed a mechanism dependent on the activation of NMDAR and independent on plasmin. *In vitro* and *ex vivo* studies also reported pro-survival effects of tPA on neurons (Liot et al., 2006; Lee et al., 2007; Polavarapu et al., 2007; Bertrand et al., 2015; Lemarchand et al., 2015), mainly anti-apoptotic effects. Also interesting, tPA was reported to attenuate zinc-induced neuronal cell death independently of its proteolytic action (Kim et al., 1999; Siddiq and Tsirka, 2004). Despite the heterogeneity of the paradigms used in these different studies, they all showed that this effect of tPA occurs independently of its proteolytic activity, with the activation of either PI3K/Akt, AMPK- or mTor-HIF-1alpha-dependent signaling pathways needed (Correa et al., 2011; Wu et al., 2012; **Figure 3B**). Two candidates have been proposed as the receptors mediating the pro-survival effects of tPA: Annexin II and EGF receptor (Siao and Tsirka, 2002; Wu et al., 2012; Bertrand et al., 2015; Lemarchand et al., 2015). The ability of tPA to convert the pro-neurotrophins (BDNF, NGF) to their active forms (Pang et al., 2004) is also a possible explanation to the pro-survival effects of tPA.

## What are the Possible Explanations of the Differential Effects of tPA on Neuronal Survival? (**Table 1**)

**Table 1:** Reported effects of tPA on challenged neurons.

Reference	Model(s)		tPA	Mechanism(s)
**Beneficial**				
Kim et al., 1999	*In vitro*: cortical cultures exposure to 300 mM zinc (mice)		Exogenous 10 μg/ml	Independently of its proteolytic action, tPA attenuated zinc-induced cell death
	*In vivo*: kainate injection (10 mg/kg) in rats		Intracerebroventricular tPA 1 mg/ml	tPA attenuated kainate seizure-induced neuronal death in the hippocampus

Flavin and Zhao, 2001	*In vitro*: OGD, 2.5 h Cultured hippocampal neurons from rats (DIV 7–10)		Exogenous 1,000 IU	tPA protects neurons from oxygen glucose deprivation (OGD) by a non-proteolytic action

Centonze et al., 2002	*Ex vivo*: striatal neurons WT and tPA –/– mice subjected to OGD		Endogenous	tPA enhanced ischemia-induced neuronal damage by facilitating apoptosis rather than necrosis

Yi et al., 2004	*In vitro*: mixed cortical cell cultures (mice) Treatment: zinc (35 μmol/l)		Exogenous 10 μg/ml	tPA attenuated zinc-induced neuronal death, independently of its proteolytic activity

Head et al., 2009	*In vitro*: primary cultures of neurons (DIV 5–21) exposed to 1.4% isoflurane for 4 h *In vivo*: 1.4% isoflurane (anesthetic mediated neurotoxicity in mice)		Exogenous 0.03–3 μg/ml	Isoflurane induced apoptosis at DIV 5 (but not DIV 14 or DIV 21) in cultured neurons tPA decreases isoflurane-induced cell death in primary cultures of neurons (DIV 5) Isoflurane-induced neurotoxicity in the developing rodent brain is mediated by reduced tPA synaptic release and enhanced proBDNF/p75NTR-mediated apoptosis

Echeverry et al., 2010	*In vitro*: cultures of hippocampal neurons (OGD conditions for 30 min (preconditioning) or not, followed 24 h later by incubation under OGD conditions for 55 min)		Endogenous (tPA KO mice) and exogenous (0–1 μM)	Treatment after OGD (early preconditionning). Beneficial effect of tPA involving a LRP1 dependent signaling pathway and independent of its proteolytic activity. Treatment 24 h after OGD (delayed preconditioning): beneficial effect of tPA via a NMDA-dependent signaling pathway (activation of pAkt), and activation of plasmin

Wu et al., 2012	*In vitro:* cultures of cortical neurons (55 min OGD and then exposed 10 min later to a second episode of hypoxia (10 min OGD, post-conditioning))		Endogenous (transgenic mice T4)	Decrease of the activation of mTor- HIFα, involving NMDAR

Wu et al., 2013a	*In vivo*: excitotoxin-induced neuronal death T4 mice and WT Intrastriatal injection of NMDA (50 mM)		T4 mice or IV 1 mg/Kg on WT mice	tPA protected the brain from excitotoxin-induced cell death Dose-dependent effect of tPA on NMDA-induced neuronal death – 5 and 10 nM beneficial – 100 at 500 nM deleterious
				(1) The neuroprotective effect of tPA was mediated by activation of synaptic GluN2A containing NMDAR via a plasminogen-independent mechanism (2) ERK activation mediated the protective effect of tPA against excitotoxin-induced neuronal death
	*In vitro:* cerebral cortical neurons (mice) NMDA induced neuronal death (50 M)		Exogenous 5–500 nM	(3) tPA activated the ERK -CREB-Atf3 pathway (4) Atf3-mediated the protective effect of tPA against excitotoxin-induced neuronal death

Wu et al., 2013b	*In vitro*: cultures of cortical neurons (OGD 55 min)		Endogenous (transgenic mice T4)	Adaptation to metabolic stress – AMPK activation involving NMDAR

Henry et al., 2013	*Ex vivo*: cortical brain slices from postnatal P10 mice		Exogenous (20 μg/mL)	tPA significantly reduced caspase-3 activity In superficial layers (less mature), tPA alone inhibited apoptosis via EGFR

**No effects**				
Vandenberghe et al., 1998	*In vitro:* spinal cords cultures of mice tPA –/– and WT (DIV 10–12) Kainate-induced death of motoneurons (20 and 100 μM for 24 h)		Endogenous	tPA did not affect the vulnerability of cultured neurons to kainite

Tucker et al., 2000	*In vivo:* primary cultures of rat cortical neurons Treatment: Aβ (16 or 25 μM) and plasminogen (30 nM)		Exogenous 10 μg/ml	tPA required plasminogen to inhibit Aβ toxicity and to block Aβ deposition Degradation of Aβ fibrils is dependent on tPA and Plg proteolytic activity

Flavin and Zhao, 2001	*In vitro:* cultured hippocampal neurons from rats (DIV 7–10) ± NMDA 10 μM		Exogenous 1,000 IU	tPA resulted in a modest exaggeration of this injury

Yi et al., 2004	*In vitro:* mixed cortical cell cultures (mice) Treatment: NMDA (30 μmol/l)		Exogenous 10 μg/ml	Calcium-mediated neuronal death was not attenuated by tPA

**Deleterious**				

Tsirka et al., 1995	*In vivo:* kainate induced neuronal death	Mouse tPA –/–	Endogenous	tPA is required to promote neuronal degeneration


		Mouse WT	120 μg tPA for 3 days (intra-parenchymal)	

Wang et al., 1999	*In vitro:* PC12 cells and primary cultures of cortical neurons (rats; DIV 12–14)		Exogenous 50 μg/ml	tPA significantly increased hemoglobin-induced cell death

Flavin and Zhao, 2001	*In vitro:* cultured hippocampal neurons on rats (DIV 7–10) ± plasminogen		Exogenous 100 IU	Proteolytic action

Nicole et al., 2001	*In vitro*: mixed cortical cultures or near-pure neuronal cultures (mice)		Exogenous 0.2–20 μg/ml	tPA failed to modify the neurotoxicity induced by the exposure to a non-NMDA agonist (kainate)
	Excitotoxicity: NMDA (10 or 12.5 μM) or 50 μM kainate Calcium imaging			The catalytic activity of tPA enhanced neuronal death induced by exposure to NMDA tPA cleaves the GluN1 subunit of the NMDAR
	*In vivo*: NMDA induced excitotoxic lesions (rats) (50 nmol)		Exogenous 3.0 μg (intra-parenchymal)	

Gabriel et al., 2003	*In vitro:* cultured cortical neurons (mice) Mixed cortical cultures of neurons and astrocytes (mice)	Apoptosis: serum deprivation (DIV 7)Nifedipine (50 μM, DIV 14) Excitotoxicity (DIV 13–14) 12.5 μM of NMDA	Endogenous	TGF-α rescued neurons from NMDA-induced excitotoxicity in mixed cultures through inhibition of tPA activity, involving PAI-1 overexpression by an ERK-dependent pathway in astrocytes

Liberatore et al., 2003	*In vivo*: kainate-induced excitotoxicity on tPA –/– and WT mice (1.5 nmol of kainate)		Exogenous 1.85 μmol/L	Infusion of tPA into tPA –/– mice restored sensitivity to kainate-mediated neurotoxicity and activation of microglia
	*In vivo*: NMDA-induced excitotoxicity in mice (50 mmol/L NMDA)		Exogenous 46 μmol/L	tPA increased the lesion volumes induced by NMDA injection into the striatum

Liot et al., 2004	*In vitro:* pure cultures of mouse cortical neurons exposed to NMDA (12.5 μmol/L)		Exogenous 20 μg/ml	Proteolytic activity

Liu et al., 2004	*In vitro*: primary neuronal cultures (mice; DIV 14) NMDA treatment–induced apoptosis in neurons		Exogenous 20 μg/ml	tPA potentiated apoptosis in mouse cortical neurons treated with *N*-methyl-D-aspartate (NMDA) by shifting the apoptotic pathway

Benchenane et al., 2005	*In vivo:* striatal excitotoxic lesions (rats; NMDA 50 nmol)		Exogenous IV 1 mg/kg	tPA potentiated excitotoxic lesions

Lebeurrier et al., 2005	*In vivo*: excitotoxic lesions in mice induced by NMDA (10 nmol in striatum or 20 nmol in cortex)		Endogenous	Overexpression of neuroserpin in the brain parenchyma might limit the deleterious effect of tPA on NMDAR-mediated neuronal death
	*In vitro*: neuronal cortical cultures from mice	Serum deprivation (DIV 7)		
	Treatment: neuroserpin (0.5–1 μM)	Excitotoxic paradigms (DIV 13–14) NMDA (12.5 μmol/l) AMPA (10 μmol/l) Calcium videomicroscopy		

Medina et al., 2005	*In vitro*: mouse neuroblastoma N2a cells; primary cultures of hippocampal neurons tPA –/– or WT (mouse)		Exogenous 20 μg/ml	tPA induced Erk1/2 activation in neurons (independently of plasmin), tau phosphorylation and promoted A-beta mediated apoptosis tPA treatments induced GSK3 activation, tau hyperphosphorylation, microtubule destabilization and apoptosis in hippocampal neurons

Benchenane et al., 2007	*In vivo* studies: Excitotoxic lesions in mice performed by injection of NMDA (10 nmol) into the striatum *In vivo* studies: permanent MCAO in mice		Exogenous 1 mg/kg	Immunization against the NTD of the GluN1 subunit of NMDAR prevented the neurotoxic effect of endogenous and exogenous tPA

López-Atalaya et al., 2007	*In vivo:* striatal excitotoxic lesions (rats; 50 nmol)		Exogenous IV 1 mg/kg	tPA increased lesion volumes induced by NMDA (+40%)

López-Atalaya et al., 2008	*In vitro:* pure neuronal cultures (mice)	Excitotoxicity (NMDA 10 μmol/L) Calcium videomicroscopy (NMDA 12.5–100 μmol/L)	Exogenous 0.3 μmol/L	Interaction of tPA with GluN1 led to a subsequent potentiation of NMDA-induced calcium influx and neurotoxicity

Wiegler et al., 2008	*In vitro*: hippocampal slices from P12 rats (OGD 30 min) Treatment: c-Jun N-terminal kinase inhibitor (XG-102; 12 nM 6 h after OGD)		Exogenous 0.9 μg/ml	Addition of tPA after OGD enhanced neuronal death in CA1 and XG-102 administration reduced neuronal death, alone or in the presence of tPA

Sun et al., 2009	*In vitro:* cultured dopaminergic neuroblasts (rat; N27 line) Treatments: aprotinine (200 KIU/ml), 𝜀-aminocaproic acid (2 mM), EGRck (Glu–Gly–Arg–CH2Cl, 100 mg/ml), FPRck (Phe–Pro–Arg–CH2Cl, 100 mg/ml), bivalirudin (20 mg/ml)		Exogenous 10–20 μg/ml	tPA induced N27 neuroblast cell death. Aprotinin and other protease inhibitors led to an inhibition of tPA-mediated neurotoxicity Aprotinin, FPRck, and EGRck directly antagonized the proteolytic activity of tPA, whereas 𝜀-aminocaproic acid inhibited the binding of tPA to lysine residues on the cell surface

Baron et al., 2010	*In vitro* study: cortical and hippocampal neurons from mice (DIV 7 or DIV 12–14). Excitotoxic neuronal death (NMDA 50 μM)		Exogenous 20 μg/ml	Catalytic tPA promoted NMDAR-induced Erk(1/2) MAPK activation tPA failed to potentiate excitotoxicity of hippocampal neurons lacking GluN2D tPA exacerbated neurotoxicity through GluN2D-containing NMDAR via Erk 1/2
	*In vivo*: excitotoxic lesions. Male Swiss mice Hippocampal or cortical bilateral injections of NMDA		Exogenous IV 10 mg/kg	

Guo et al., 2011	*In vitro:* mouse cortical neurons (DIV14) Neuronal apoptosis model		Exogenous 20 μg/ml	The anticoagulant factor protein S (PS) protects mouse cortical neurons from tPA/NMDA induced injury. PS blocks the extrinsic apoptotic cascade

Jullienne et al., 2011	*In vitro*: cortical and hippocampal neurons (mice; DIV 12–13) Excitotoxic neuronal death: NMDA (10 μM) Treatment: UBP145 (0.2 μM)		Exogenous 20 μg/mL	tPA increased NMDA-mediated neurotoxicity in cortical neuronal cultures but not in hippocampal neuronal cultures UBP145 had no effect on NMDA-mediated neurotoxicity in hippocampal neurons but prevented tPA-induced potentiation of NMDA-mediated neurotoxicity in cortical neurons
	*In vivo:* cortical excitotoxic lesions NMDA (mice; 2.5 nmol) Treatment: UBP145 (0.05 nmol)		Exogenous IV 10 mg/kg	Inhibition of GluN2D-containing NMDAR with UBP145 can fully prevent the pro-excitotoxic effect of intravenously administered tPA

Rodríguez-González et al., 2011	*In vitro:* primary mixed cortical cell cultures from rats (OGD 150 min)		Exogenous 5 mg/mL	Treatment with tPA after OGD increased LDH release, active MMP-9, MCP-1, and MIP-2 Treatment with neuroserpin after OGD decreased LDH release and active MMP-9

Roussel et al., 2011	*In vitro:* primary cultures of cortical neurons (mice; DIV 10) Excitotoxicity induced by 10 μM NMDA Treatment: HMGB-1 0.3 μM		Exogenous 0.3 μM	HMGB-1 reversed the pro-neurotoxic effect of tPA HMGB-1 prevented tPA from potentiating NMDA-evoked Ca^2+^ influx

Ma et al., 2012	*In vitro:* cultures of cortical neurons (rats; OGD/R) Treatment: neuroserpin		Endogenous	Neuroserpin protected neurons against OGD/R. mainly by inhibiting tPA-mediated acute neuronal excitotoxicity

Montagne et al., 2012	*In vitro:* cortical cultures of neurons from mice (DIV 12–13) Treatment: memantine (1–10 μmol/L)	Excitotoxicity NMDA (10 μmol/L) OGD (30 min) Calcium videomicroscopy NMDA (50 μmol/L)	Exogenous 0.3 μmol/L	Memantine prevented the potentiation of excitotoxic neuronal death induced by rtPA Memantine prevented rtPA-exacerbated calcium influx through activated NMDAR
	*In vitro:* cultures of cortical neurons from mice (DIV 15–16) Excitotoxic neuronal death: NMDA 50 μM		Exogenous 0.3 μM	In contrast to WT tPA, tPA mutants including deletion of the kringle 2 domain and point mutation of the LBS-containing kringle 2 domain did not promote NMDAR-mediated neurotoxicity

Parcq et al., 2012	*In vitro*	Excitotoxicity induced by exposure of cortical neurons to NMDA (mice; 50 μM) at DIV 14	Exogenous 0.3 μM	sc-tPA promoted NMDAR-mediated neurotoxicity through its proteolytic activity, tc-tPA did not sc-tPA promoted both NMDA-induced calcium influx and Erk (½) activation, tc-tPA did not
		NMDA-induced calcium influx recorded from cultured cortical neurons (mice; DIV 12–14) exposed to NMDA (50 μM)		
	*In vivo*	NMDA-induced excitotoxic brain lesions (NMDA 10 mM)	Exogenous 45 μM	

Henry et al., 2013	*Ex vivo:* cortical brain slices from postnatal P10 mice		Exogenous 20 μg/mL	In deeper layers (more mature), tPA was associated with glutamate-promoted neuronal necrosis

Omouendze et al., 2013	*In vivo:* excitotoxic insult by intra-cortical injection of Ibotenate in rats PAI-1 or tPA –/– or WT *Ex vivo*: brain sections		Endogenous or exogenous 20 μg/ml	Neonatal brain lesions

### Are Target Receptors the Explanations?

In the brain parenchyma, pro-survival and pro-neurotoxic effects of tPA have been shown to involve key receptors/pathways, including NMDAR (Nicole et al., 2001), LRP-mediated PSD95 activation (Martin et al., 2008), annexin-II (Siao and Tsirka, 2002), and EGF receptor (Liot et al., 2006; Lemarchand et al., 2015). Focusing on NMDARs, the fact that tPA induces toxic or protective effects could also depend on the different subtypes of GluN subunits involved, and/or their location (synaptic versus extrasynaptic; Paoletti et al., 2013). For instance, based on the current literature, it could be postulated that exogenous tPA could promote neurotoxicity on cortical neurons by activating extrasynaptic GluN2D-containing NMDARs (Baron et al., 2010; Jullienne et al., 2011; Montagne et al., 2012), but could lead to a neuroprotective effect by activating synaptic GluN2A-containing NMDARs (Wu et al., 2013a; **Figure 3B**). Several studies also propose that the neuroprotective activity of tPA, even in a paradigm involving NMDARs, is NMDAR-independent (Correa et al., 2011), independent of its proteolytic activity (Liot et al., 2006). In a model of apoptosis induced by serum deprivation (Liot et al., 2006) or when subjected to OGD, the neuroprotective effect of tPA is mediated by an activation of either EGFR (Correa et al., 2011; Bertrand et al., 2015; Lemarchand et al., 2015) or annexin II (Lee et al., 2007). Whether LRP is also involved is still under debate, again dependent on the paradigm used (Martin et al., 2008). Up to now, it is not clear how these different receptors contribute to the differential effects of tPA in neuronal survival. Additional studies are needed including investigations about possible crosstalks between these different receptors.

### Are Protocols of Neuronal Injury the Explanation?

Type plasminogen activator-dependent over-activation of NMDARs has been proposed as a mechanism that could mediate both neuroprotective (Wu et al., 2013b) and neurotoxic (Baron et al., 2010) effects of tPA (**Figure 3B**). This discrepancy may be explained by the use of different models to induce neuronal death, either pure NMDAR-mediated excitotoxicity (Baron et al., 2010) or oxygen glucose deprivation (OGD; Wu et al., 2013b). Whether OGD induces excitotoxicity and/or apoptosis is not well documented and might depend on the severity/duration of the stress. Pathways such as autophagy or endoplasmic reticulum stress may also occur (Badiola et al., 2011; Shi et al., 2012). Another explanation could be the use of differential strategies to block tPA-induced potentiation of NMDAR signaling, MK-801 as a broad irreversible antagonist of NMDARs on one hand (Terro et al., 2000) and an antibody previously characterized to specifically prevent the tPA-dependent potentiation of NMDARs signaling without affecting their basal activity (Benchenane et al., 2007; Macrez et al., 2010) on the other hand. It is interesting to note that either over-activation and blockage of NMDARs are neurotoxic, the first one leading to excitotoxic neuronal death (Nicole et al., 2001), the second one inducing apoptosis (Mattson and Duan, 1999; Henry et al., 2013).

### Is Neuronal Maturity an Explanation?

To discuss the differential impact of tPA on neuronal survival, how neurons are mature is also an important issue including whether experiments were performed *in vitro* (neuronal cultures performed from E16 embryo and maintained different times *in vitro*, 5–14 days (Buisson et al., 1998; Samson et al., 2008), *ex vivo* (hippocampal slices harvested at P3 and maintained different times *in vitro*; Lemarchand et al., 2015) or *in vivo* (young versus aged animals; Roussel et al., 2009). For example, it was well-demonstrated that mouse primary cultures of cortical neurons become sensitive to NMDA-induced neuronal death only after 10 days *in vitro*, an effect potentiated by exogenous tPA (Launay et al., 2008). At early times (days *in vitro*), they require trophic factors contained in the culture media (serum) to survive (Hetman et al., 2000; Terro et al., 2000). When removed, serum deprivation led to neuronal apoptosis with a protective effect of exogenous tPA (Liot et al., 2006). Type of neurons may also be critical, with neurotoxic effects of tPA mainly described in cortical neurons (Nicole et al., 2001) or Purkinje neurons (Cops et al., 2013; Li et al., 2013; **Figures 3B,C**). The protective effect of tPA was described on hippocampal neurons (Flavin and Zhao, 2001; Echeverry et al., 2010; Lemarchand et al., 2015; **Figure 3A**) and on cortical neurons (Liot et al., 2006; Wu et al., 2013a; **Figure 3B**).

### Does the Origin of tPA (Endogenous vs. Exogenous) make the Difference?

Another important point of discussion is to know whether exogenous and endogenous tPA have differential effects on neuronal survival. The most recent literature in this field demonstrates that endogenous tPA displays neuroprotective activities (Wu et al., 2013a; Lemarchand et al., 2015) and exogenous tPA is neurotoxic (Parcq et al., 2012). Nevertheless, using tPA deficient mice, exogenous tPA may also protect hippocampal neurons subjected to OGD (Lemarchand et al., 2015). These data suggest that tPA (exogenous or endogenous) may have either pro-neurotoxic or pro-survival effects depending of the type of stress paradigms used and/or the type of neurons. Thus, whether experiments are performed on wild type neurons, tPA deficient neurons, tPA over-expressing neurons, *in vitro* and *in vivo*, is important to understand the impacts of tPA on neuronal survival (Tsirka et al., 1995; Wang et al., 1998; Nicole et al., 2001; Liot et al., 2006; Echeverry et al., 2010; Wu et al., 2013a).

## What about the Level of tPA?

Some authors suggest that low levels of tPA are neuroprotective (Wu et al., 2013a), either exogenous (Baron et al., 2010) or produced by stressed cells (Lemarchand et al., 2015). In contrast, high levels of tPA (mainly exogenous) are neurotoxic (Nicole et al., 2001; Parcq et al., 2012).

### Finally, Why not the Form of tPA?

There is so far only one study which discriminated tPA isoforms in the context of neuronal survival, with a clear evidence that sc-tPA is the only one capable to activate NMDAR and to promote excitotoxicity (in mouse cortical neurons subjected to NMDA exposure) both *in vitro* and *in vivo* (Parcq et al., 2012; Bertrand et al., 2015). It is thus interesting to note, that complexes formed between sc-tPA and neuroserpin (NSP) were reported more stable than those formed between tc-tPA and NSP, with no differences when complexes are formed with PAI-1 (Barker-Carlson et al., 2002). Whether conversion of sc-tPA into tc-tPA (by plasmin like activity) may influence the functions of tPA on neuronal survival, especially in the context of brain injuries, need to be investigated.

## Conclusion

Depending on the study, endogenous tPA was reported as deleterious or beneficial for neurons. Although it is difficult to reconcile these findings, some propose that tPA is neuroprotective at low levels, but neurotoxic at higher levels. Assays of extracellular levels of tPA under specific conditions should be provided to support this hypothesis. Undoubtedly, the target involved is also a key trigger in the effect of tPA. In general, the pro-survival effects of tPA are independent on its proteolytic activity involving, interconnected or independently, EGF receptors, annexin II, PI-3 kinase-, AMPK-, mTor-HIF-1alpha-dependent signaling pathways. In the adult, the neurotoxic effects of tPA seem to be dependent on its proteolytic activity, targeting either plasminogen, NMDARs, components of the extracellular matrix, inflammatory mediators, and/or other proteases. However, indirect neurotoxicity might also occur via a non-proteolytic activation of microglia (Siao and Tsirka, 2002). For now, there is no clear clinical data to determine, in human, whether tPA is neurotrophic or neurotoxic and in what conditions. Additional studies are needed to understand further the possible differential functions of tPA on neuronal survival. To address this question, we should consider the different isoforms of tPA (type I sc-tPA, type I tc-tPA, type II sc-tPA, and type II tc-tPA), the possibility that tPA may activate its substrates and/or receptors with differential affinities and that these substrates and/or receptors could be differentially expressed in cortical versus hippocampal neurons depending on their maturity.

## Conflict of Interest Statement

The authors declare that the research was conducted in the absence of any commercial or financial relationships that could be construed as a potential conflict of interest.
